# A novel single-cell RNA-sequencing approach and its applicability connecting genotype to phenotype in ageing disease

**DOI:** 10.1038/s41598-022-07874-1

**Published:** 2022-03-08

**Authors:** Orr Shomroni, Maren Sitte, Julia Schmidt, Sabnam Parbin, Fabian Ludewig, Gökhan Yigit, Laura Cecilia Zelarayan, Katrin Streckfuss-Bömeke, Bernd Wollnik, Gabriela Salinas

**Affiliations:** 1 NGS- Core Unit for Integrative Genomics, Institute of Human Genetics, University Medical Center Göttingen, Göttingen, Germany; 2 Institute of Human Genetics, University Medical Center Göttingen, Göttingen, Germany; 3 Institute of Pharmacology and Toxicology, University Medical Center Göttingen, Göttingen, Germany; 4 Clinic of Cardiology and Pneumology, University Medical Center Göttingen, Göttingen, Germany; 5 DZHK (German Centre for Cardiovascular Research) Partner Site Göttingen, Göttingen, Germany; 6 Institute of Pharmacology and Toxicology, University of Würzburg, Würzburg, Germany; 7 Cluster of Excellence “Multiscale Bioimaging: From Molecular Machines To Networks of Excitable Cells” (MBExC), University of Gottingen, Gottingen, Germany

**Keywords:** Computational biology and bioinformatics, Genetics, Genomic analysis, Sequencing

## Abstract

Single cell multi-omics analysis has the potential to yield a comprehensive understanding of the cellular events that underlie the basis of human diseases. The cardinal feature to access this information is the technology used for single-cell isolation, barcoding, and sequencing. Most currently used single-cell RNA-sequencing platforms have limitations in several areas including cell selection, documentation and library chemistry. In this study, we describe a novel high-throughput, full-length, single-cell RNA-sequencing approach that combines the CellenONE isolation and sorting system with the ICELL8 processing instrument. This method offers substantial improvements in single cell selection, documentation and capturing rate. Moreover, it allows the use of flexible chemistry for library preparations and the analysis of living or fixed cells, whole cells independent of sizing and morphology, as well as of nuclei. We applied this method to dermal fibroblasts derived from six patients with different segmental progeria syndromes and defined phenotype associated pathway signatures with variant associated expression modifiers. These results validate the applicability of our method to highlight genotype-expression relationships for molecular phenotyping of individual cells derived from human patients.

## Introduction

In the field of genomic medicine, contemporary single-cell sequencing methods are focusing on the characterization of individual cells. Recent advances in integrated robotic systems and molecular barcoding have made the transcriptional profiling of thousands of individual cells cost-effective^1,2^.

Available integrated systems for single-cell applications differ in their sensitivity, specificity and throughput. They also differ in other critical experimental parameters such as the detection of doublet and debris, limitations in capturing rate due to cell sizing or cell morphology and documentation or visualization of the captured single cells.

The most critical step for obtaining transcriptome and genome information from individual cells is the single cell isolation, and thus it is necessary to distinguish between methods with low- or high-throughput for the collection of single cells. Low-throughput approaches such as limiting dilution, micromanipulation and laser capture microdissection (LCM) are time-consuming and exhibit limitations in the capture of rare cells^2,3^. Flow-activated cell sorting (FACS) is a commonly used strategy for isolating highly purified single cells. The potential limitations of these techniques include the requirement of large starting volumes (difficulty in isolating cells from low-input numbers < 10,000) and the need for monoclonal antibodies to target proteins of interest leaving novel cell populations unexplored^2–4^.

Recently, integrated systems for both single cell collection and downstream experiments, particularly those related to single-cell NGS applications, have become commercially available. In particular, the microdroplet-based microfluidics technology such as those used by Drop-Seq and 10× Genomics Chromium platform, uses a gel bead coated with oligonucleotides^5,6^. A further well-established integrated system for the performance of single-cell RNA sequencing (scRNA-seq) is the ICELL8 platform. Previously, several studies explored the utility of this platform for single-cell sequencing in the cardiac field, due to the fact that cardiomyocyte size limits the use of drop-seq and other platforms^7,8^.

Several groups performed comparisons between different scRNA-seq platforms and chemistry^9–12^. However, these comparisons utilized custom-build drop-seq platforms and/or reaction chemistry which can potentially be a hurdle for investigators new to the scRNA-seq field^13–15^.

In this paper, a combination of two instruments, CellenONE X1 (CellenONE) and ICELL8 cx Single-Cell System (ICELL8), was implemented and validated as a new method for scRNA-seq. CellenONE allows the selection of highly purified single cells based on cell sizing, cell morphology or by using one or more fluorescence markers prior to sample processing and sequencing.

Moreover, by using this approach we improved the cell capturing efficiency from 1200 to 1400 cells usually obtained by the ICELL8 system to > 3300 cells when combining both instruments. Here we report a novel high-throughput single-cell RNA-seq approach with full coverage of transcripts using the SMART-Seq technology, demonstrating the molecular characterization of mutational and transcriptional heterogeneity in dermal fibroblasts derived from six patients with different segmental progeria syndromes. In addition to the causative mutations previously identified in these patients^16,17^, we performed regulon analysis to identify interactions of genetic alterations that can be held responsible for the phenotypic heterogeneity.

## Results

### Method overview of integrated platforms for scRNA-sequencing

**Figure 1:**
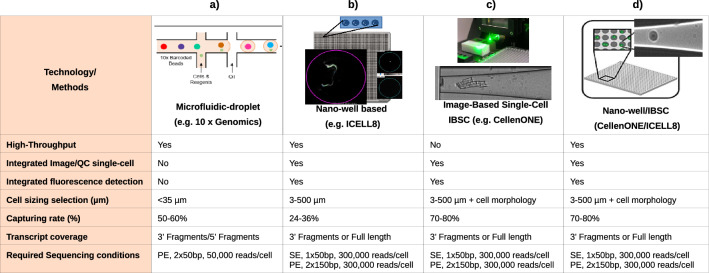
Technical information of state-of-the-art integrated platforms for single-cell RNA-Sequencing. Methods used in each of the platforms for single-cell isolation, barcoding, chemistry and sequencing. (**a**) the 10× Genomics Chromium (**b**) the ICELL8 cx Single-Cell system; ICELL8 (**c**) the CellenONE X1 system, CellenONE and (**d**) advantages of the combined CellenONE X1 and iCELL8 cx Single-Cell systems, CellenONE-ICELL8.

The most used state-of-the-art integrated platforms for single-cell collection are illustrated in Fig. 1. All platforms have an integrated system that includes cells dispensing, conversion of RNA into cDNA (RT) and library preparation for sequencing. The 10 × Genomics Chromium platform (Fig. 1a) is able to process thousands of individual cells in a fully automated manner. However, this platform exhibits some limitations e.g. cell sizing (< 35 µm) and the lacking of capability for evaluating the quality of single cells based on imaging features prior to sequencing, discarding damaged cells that potentially generate artificial cell clusters. Moreover, the chemistry used is based on 3’ or 5’ fragment, which is not optimal for the investigation of mutational heterogeneity in single cells.

ICELL8 (Fig. 1b) is a well-established platform for scRNA-Seq that can select living cells, while discarding dead cells and doublets by imaging^7,8,18–21^. The chemistry used in this study for single-cell cDNA synthesis is the full-length SMART technology (full coverage of transcripts). Previously published datasets based on single cells sequenced with Smart-seq chemistry have been used for cell atlas analyses^18,19^. The instrument exhibits a low cell-capturing rate that varies between 24 and 36% from 5,184 cells. Depending on the cell system used, a range of 800 to 1,400 cells can be analyzed in one experiment (one chip with 5,184 nano-wells). The advantages of ICELL8 include flexible chemistry for transcript coverage (3’ end fragment or full-length approaches), flexible sequencing mode selection choosing between single-end (SE) or paired-end (PE) mode, compatible for both living as well as fixed cells with a large range of sizes (3 µm to 500 µm in diameter) and dispensing of cells with abnormal morphology e.g., Schwann cells that exhibit axonic regrowing forms and sizing of 200–500 µm, as we show in this paper for the first time (Fig. 1b and Supplementary Fig. 1).

CellenONE offers an excellent imaging system for cell collection. The technology, which is based on Image Based Single Cell Isolation (IBSCIT), offers high-definition optics for cell visualization and can isolate cells over a wide size range. Here we demonstrated, the dispensing and documentation of cardiomyocytes (whole cells) with a cylindrical shape (35–120 µm) using this system (Fig. 1c)^20^. One disadvantage this instrument has is its inability to process thousands of cells simultaneously for sequencing in a fully automated manner.

In order to improve the capturing rate of ICELL8 and to simultaneously process more than 5,000 cells to libraries for sequencing, we combined both instruments (Fig. 1b and c) by collecting the cells from CellenONE directly into a Takara ICELL8 5,184 nano-well chip (Fig. 1d). The system allows the isolation of living or fixed cells and the performance of an analysis on nuclei or whole cells without limitations regarding cell sizing or cell morphology. Moreover, the exclusion of cell doublets and debris can be done by defining the parameters during cell collection or by cell inspection after dispensing. Each collected cell is reported and documented, allowing an additional check for cell quality before sequencing. The ICELL8 5,184 nano-well chip was processed for RT and library preparation fully automated in the ICELL8 system.

### Validation of the novel scRNA-seq approach using human dermal fibroblasts

In order to evaluate the composite CellenONE-ICELL8 approach with respect to the well-established ICELL8 platform, we used dermal fibroblasts from a unique cohort from six progeria patients (GOE1360, GOE1309, GOE800, GOE615, GOE486 and GOE247) and two age-matched, healthy control individuals (GOE1303 and GOE1305). Both methods were compared using the same chemistry, the full-length SMART technology.

**Figure 2:**
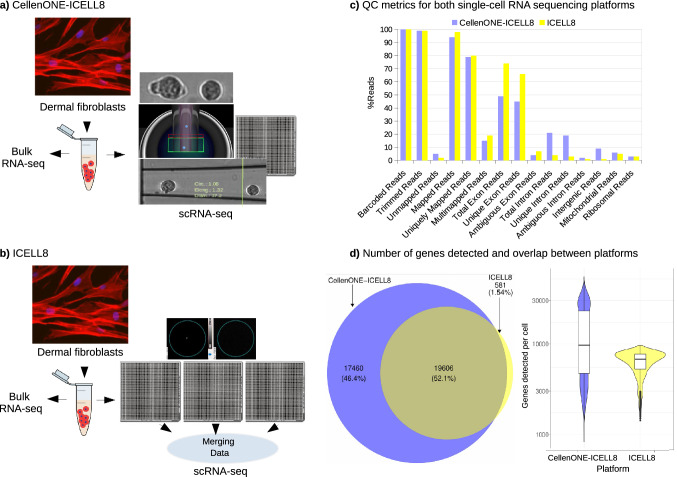
Study design for the scRNA-Seq approaches validation. (**a**) Experimental design for the CellenONE-ICELL8 Single-Cell System combination. Only one ICELL8 nano-well chip was used for all eight dermal fibroblasts patient samples. (**b**) Experimental design for the ICELL8 Single-Cell System. A total of three (3) ICELL8 nano-well chips were used for all eight dermal fibroblasts patient samples. (**c**) Bar plot displaying proportions of reads left in both single-cell methods after each step of the analysis. (**d**) Venn diagram displaying the number of genes in QC-filtered data from both approaches and the violin plot shows the number of QC-filtered genes expressed in individual cells in both approaches.

Initially, CellenONE dispensed single cells from the eight dermal fibroblasts samples to a single Takara ICELL8 5,184 nano-well chip. The dispensing parameters used for human dermal fibroblasts cells were based on sizing (20–60 µm in diameter, elongation and circularity of 1–1.5 µm, see Fig. 2a). Following first quality control based on single cell collection, we discarded 123 (doublets) of 5,184 cells. After sequencing and quality-control (QC) filtering, a total of 3,135 cells were processed for data analysis (Fig. 2a, Table 1). For the ICELL8 single-cell approach, cells from the same samples were dispensed in three ICELL8 5,184 nano-well chips obtaining 3,129 cells after data merging and sequencing QC filtering (Fig. 2b, Table 1). Furthermore, the remaining cells from the scRNA-seq experiments were collected for bulk RNA-seq as shown in Figs. 2a and b.

**Table 1:** Number of cells dispensed and selected for sequencing after QC-filtering data set (CogentDS).

	GOE1303	GOE1305	GOE1309	GOE1360	GOE247	GOE486	GOE615
**Samples CellenONE-ICELL8**							
No. of cells dispensed	576	576	576	576	576	576	576
No. of cells Post-QC	387	295	298	537	479	195	218
Total cells Post-QC	3135						
**Samples ICELL8**							
No. of cells dispensed	436	364	485	356	446	477	350
No. of cells Post-QC	423	323	411	280	386	445	317
Total cells Post-QC	3129

**Table 2:** Read parameters of the two scRNA-seq approaches CellenONE-ICELL8 and ICELL8 (CogentDS).

QC-Parameters	CellenONE-ICELL8	ICELL8
Samples	8	8
No. of cells	5184	3616 cells
No. of cells Post-QC	3135	3129 cells
Total Reads	1.67G	1.01 G
Barcoded Reads	1.61G	985 M
Fraction Barcoded Reads	0.96	0.98
Barcodes Identified	5184 cells	3616 cells
Reads per Barcode	309.84 K	272.54 K
Barcoded Reads	1606229682 (100%)	985522031 (100%)
Trimmed Reads	1601535800 (99.71%)	982927397 (99.74%)
Unmapped Reads	86672148 (5.4%)	16885303 (1.71%)
Mapped Reads	1514863652 (94.31%)	966042094 (98.02%)
Uniquely Mapped Reads	1270205342 (79,08%)	778278333 (78.97%)
Multimapped Reads	244658310 (15,23%)	187763761 (19.05%)
Total Exon Reads	788938677 (49,12%)	727757440 (73.84%)
Unique Exon Reads	714643829 (44.49%)	654119292 (66.37%)
Ambiguous Exon Reads	74294848 (4.63%)	73638148 (7.47%)
Total Intron Reads	337929018 (21.04%)	37383326 (3,79%)
Unique Intron Reads	300416455 (18,7%)	32630018 (3.31%)
Ambiguous Intron Reads	37512563 (2.34%)	4753308 (0.48%)
Intergenic Reads	143337647 (8.92%)	13137567 (1.33%)
Mitochondrial Reads	93167354 (5.8%)	57107478 (5.79%)
Ribosomal Reads	30369153 (1.89%)	29861198 (3.03%)

It is important to note, that the batch effect observed by merging data from three ICELL8 5,184 nano-well chips dispensing the same samples was very low (Supplementary Fig. 2), suggesting that a more sensitive method with deeper sequencing can mitigate the variation to a large degree of cells.

The datasets generated from both approaches were comparable in terms of numbers of total reads (1.01G in ICELL8 to 1.67G in the composite) and reads per barcode (272 K in ICELL8 to 309 K in the composite). Interestingly, when comparing both approaches, the dataset generated in the composite method showed a higher percentage of intronic and intergenic reads than ICELL8 (Fig. 2c, Table 2). Furthermore, across all samples investigated, the portion of non-coding RNAs detected by the CellenONE-ICELL8 approach, especially the detection of long non-coding RNAs (lncRNAs), was significantly higher than those detected in ICELL8 system alone were. For example, lncRNAs in sample GOE1309 compose 29% of the expressed genes in the composite approach, and only 16% of the expressed genes in ICELL8 (Table 3). This suggests that while the novel approach results in sufficient amount of exonic reads, it may also provide additional insight into the utility of intergenic sequences and introns (for instance intronic retention) on an individual cell level.

Comparing the QC-filtered transcribed genes from both methods has shown that 52% of all genes overlapped between the approaches, 1.5% were ICELL8-specific and 46% were specific in the composite approach (Fig. 2d). This result demonstrates that the novel approach can reproduce sequencing data generated by ICELL8, and has the advantage of providing additional genetic information with increased coverage of more genes.

### Correlations of gene expressions between single-cell platforms and bulk RNA-seq

**Figure 3:**
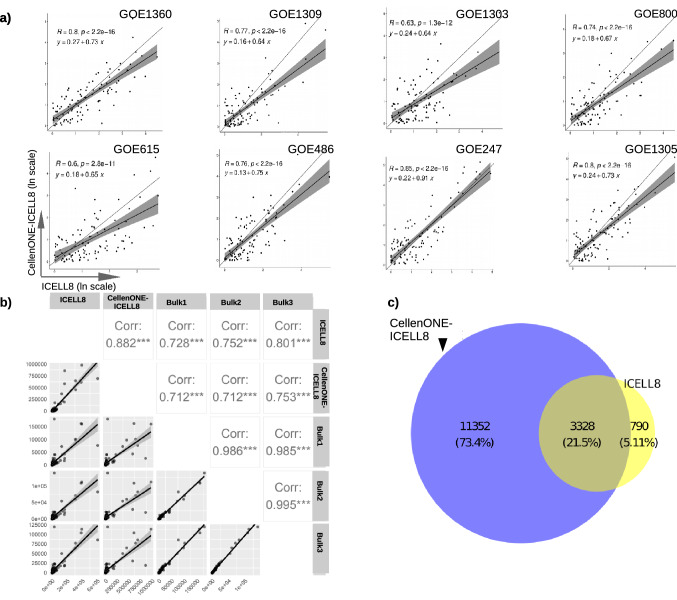
Correlations of scRNA-Seq methods and bulk RNA-seq. (**a**) Natural-log-scaled ranks of top 100 markers in each sample from both single-cell approaches, including a regression line with 95% confidence interval (in gray) and the overall correlation coefficient R. (**b**) Correlations of normalised expressions of the top 100 markers in sample GOE247 between both single-cell and bulk RNA-seq, with the correlation coefficients indicated in the upper triangle. (**c**) Venn diagram of all markers detected for sample GOE247 in both approaches.

To evaluate similarities in gene expressions between the single-cell approaches, markers were detected for each sample in each dataset, extracting the 100 top-ranked markers and calculating the correlation between their ranks (Fig. 3a). The correlations appeared high, ranging from 0.6 to 0.85 between the methods, providing additional evidence of the similarity in gene expressions, even across different samples.

The overlap of all markers between ICELL8 and CellenONE-ICELL8 was then determined for each sample and shown in Fig. 3c and Supplementary Fig. 4. The CellenONE-ICELL8 approach shows many additional unique markers compared to ICELL8, indicating high sensitivity on gene detection by the performance of the new approach and a significant overlap between both methods.

Finally, we performed a correlation analysis between the single-cell approaches and bulk RNA-seq data to demonstrate their comparability. Normalized pseudo-counts from the single-cell methods were correlated with the normalized counts from the bulk RNA-seq data using the previously mentioned top 100 markers. We observed for sample GOE247, GOE486 and GOE1309 high correlations between bulk samples and CellenONE-ICELL8 ranged from 0.71 to 0.83 (Fig. 3b and Supplementary Fig. 3). The rest of the samples show low or middle correlations. The differences reflect the efficiency of library preparation and data quality when starting bulk RNA-seq with low amounts of cells.

### Determination of the transcriptional heterogeneity in accelerated ageing phenotypes

In order to validate the scRNA-seq approach and to shed light on the transcriptional signatures, we used the unique patient cohort, all representing premature ageing phenotypes. These samples have been previously described from gene panels and whole exome sequencing, reporting causative mutations^16,17^. To evaluate transcriptional heterogeneity, we searched for genes expressed selectively in each of the samples that were described previously on cellular hallmarks of ageing driving physiological and pathological ageing processes, such as genomic instability, telomere attrition, epigenetic alterations, loss of proteostasis, mitochondrial dysfunction, deregulated nutrient sensing, stem cell exhaustion, cellular senescence and altered intercellular communication^22,23^.

**Figure 4:**
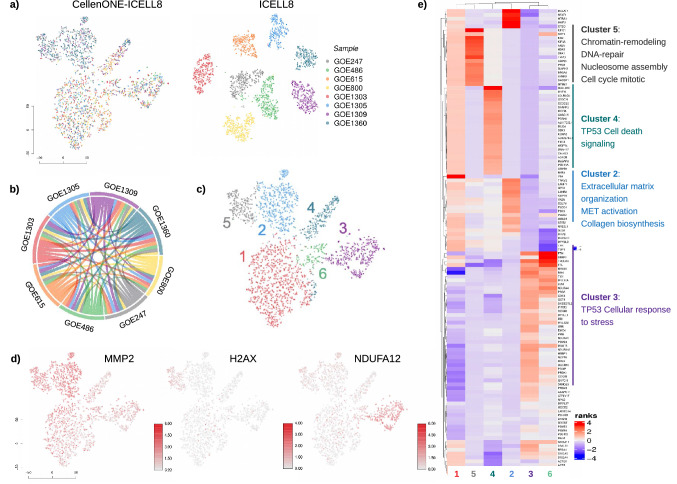
Analysis of transcriptional heterogeneity of dermal fibroblast derived from patients using the novel platform. (**a**) t-SNE of the ICELL8 and CellenONE-ICELL8 experiments. (**b**) Circo plot showing connection of top 100 marker genes of the samples in both ICELL8 and CellenONE-ICELL8 platforms. (**c**) Unsupervised t-SNE of the CellenONE-ICELL8 approach. (**d**) t-SNE showing most prominent markers for different clusters. (**e**) Heatmap of rank scores of the top 30 markers in each unsupervised cluster, including enriched pathways.

For dimensionality reduction and data visualisation, we performed the t-SNE algorithm as implemented in CogentDS (v1). To evaluate transcriptional events regarding samples in both scRNA-seq methods, we analysed cell groups based on their sample association (Fig. 4a) and performed, in parallel, unsupervised graph-based clustering of cells based on their gene expressions (Fig. 4c).

The unsupervised clustering displayed six clusters with the most significant differences in gene expression signatures between each cluster (Fig. 4c and d). The gene profiles evaluated for the clusters were associated to specific markers of dermal fibroblasts patient samples and were found to be related to pathogenesis of different ageing disorders (Fig. 4d and e). It is important to note, that several markers appear to be expressed in all samples, indicating transcriptional events that are commonly expressed within all samples. This is reflected in Fig. 4b, showing that half of the top 100 markers are expressed across investigated samples, including the controls.

We next compared cluster 1 which predominantly represents the control samples GOE1305 and GOE1303, to all other clusters related to ageing disorders. Starting with cluster 2, markers related to extracellular matrix organization, MET activation, and collagen biosynthesis (MMP2, P3H3, PLOD1, and LAMB2) were found to be upregulated in comparison to Cluster 1. Cluster 3 shows the over-expression of markers for cellular response to stress and TP53 regulating metabolic genes (NDUFA4, UBB, RPS18, HSBP1, COX4l1, ATP6V1F, TXN, PRDX1, PRDX2, PSMB6, RPL3, COX5B, and ANAPC11) when comparing to controls. Cluster 4 exhibits over-expression of markers associated with cell death signalling via NRAGE, NRIF, NADE, cell death receptor signalling pathway (MCF2L, OBSCN) and activation of metabolic gene expression (ACACB). Notably, cluster 5 shared regulation of several genes with cluster 2 which represented mainly patient samples GOE1360 and GOE247. For cluster 2 and 5, we found upregulation of genes related to chromatin-remodelling, DNA-repair, nucleosome assembly, cell cycle mitotic/checkpoints, deposition of new CENPA-containing nucleosomes, and signalling by Rho GTPases e.g., CDT1, KNL1, TMPO, GTSE1, H2HX, BRCA2, and SKA1. Based on those differing markers found for the different clusters, it is possible to display genes that, while they may not be markers for all cells within a sample, they do show particular patterns that can be linked with different progeria phenotypes.

### Variant-associated expression signatures in accelerated ageing phenotypes

We next investigated the extent to which additional variant heterogeneity in putative driver genes could be associated with transcriptional heterogeneity for ageing processes in each case. It is important to note that this analysis was done in a hypothesis-free way, not taking into account the underlying primary genetic cause and mutation of the ageing phenotype, and thereby, being able to search for additive and overlapping variant signatures and expression profiles associated with general aspects of premature ageing.

In order to evaluate how genetic regulation drives specific ageing phenotypes, we searched for patient-specific variants or non-uniformly distributed variants expressed in each of the six patients with different segmental progeria syndromes.

We first combined all variant calling files (VCFs) from both single-cell and bulk RNA-seq data, initially detecting variants in each of the scRNA-Seq samples. Most of the variants were found uniformly distributed, suggesting common effects across all samples related to ageing disease (SNPs and INDELS). Because the detection of SNPs on the scRNA-seq produces many false positives and many of the variants exhibited relatively low depth (1 or 2 reads with a variation), we selected additional criteria to label “mutant cells” considering filtering based on variant- and total depth.

To optimize depth cutoffs, the quartile distributions of the variant and total depths were calculated for each sample. Variants were filtered for variant depths > 4 (i.e., number of reads containing the variant base) and total depths > 7 (i.e., number of all reads at variant position) in the scRNA-seq data. All variants reported in this study have later been corroborated by the bulk RNA-seq data while filtering variants in the bulk data for variant depths > 6 and total depths > 10. This resulted in 756 variants for GOE1309, 94 variants for GOE1360, 254 variants for GOE247, 345 variants for GOE486, 273 variants for GOE615 and 234 variants for GOE800 (Supplementary Table 1).

We next evaluated markers whose regulation on expression are influenced by one or more variants by applying a regression-based method^24^. Table 4 described the variants selected for each of the six progeria samples, exhibiting a non-uniformly distributed mutation pattern. All six variants are non-synonymous mutations showing changes in the protein sequence, five of them were found to be reported on the dbSNP and four of them were found to be located on mitochondrial genes.

**Table 3:**
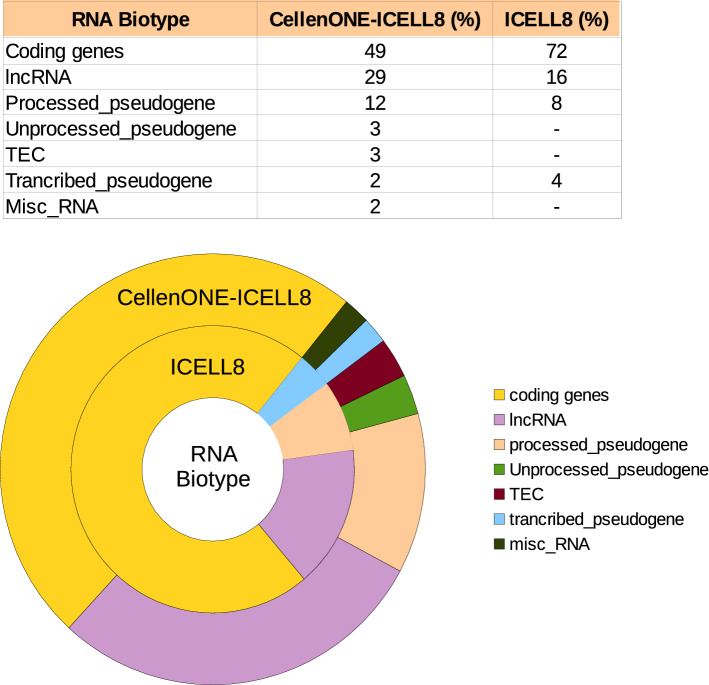
RNA-Biotype percentages identified in sample GOE1309 using the CellenONE-ICELL8 and ICELL8 methods.

**Table 4:** Overview of mutations detected in the scRNA-seq and bulk RNA-seq.

Sample	Gene	dbSNP	Chr	Position	Genotype	Codon change	Protein change	Variant name
GOE615	DAXX	rs146304558	6	33,320,019	1/1	c.1457C > G	p.Ala486Gly	DAXX^A486G^
GOE247	MRPL34	rs201529220	19	17,306,321	0/1	c.221C > T	p.Ala74Val	MRPL34^A74V^
GOE800	MT-CO1	rs202216551	M	6267	1/1	m.6267G > A	p.Ala122Thr	MT-CO1^A122T^
GOE1309	MT-ND4	rs2853494	M	11,641	1/1	m.11641A > G	p.Ile294Met	MT-ND4^I294M^
GOE486	MT-ND5	NA	M	12,557	1/1	m.12557C > T	p.Thr74Ile	MT-ND5^T74I^
GOE1360	MT-CYB	rs193302994	M	15,452	1/1	m.15452C > A	p.Leu236Ile	MT-CYB^L236I^

**Figure 5:**
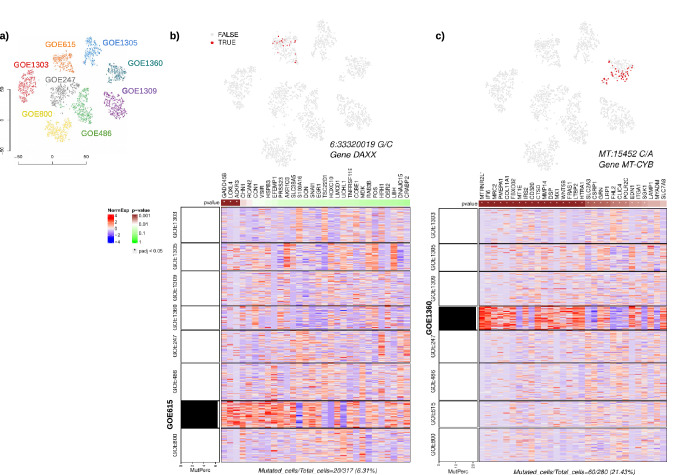
Analysis of mutational heterogeneity in dermal fibroblast derived patients. (**a**) tSNE plot showing cells classified according to their sample affiliation. (**b**) t-SNE projection showing non-uniformly distributed DAXX^A486G^ variant-expressing cells in patient GOE615 in red and the regulon for variant-dependent genes. The heatmap displays the scaled, normalized gene expressions of the top 30 markers with highest adjusted* p* values from the regression of the gene expressions to the mutation rate (*p* values shown in top bar), and a bar graph on the left shows mutant cell fraction in each cluster labelled to the left of the heatmap. (**c**) t-SNE projection showing non-uniformly distributed MT-CYB^L236I^ variant-expressing cells in GOE1360 in red and the regulon for variant-dependent genes, with a bar graph showing mutant cell fraction in each cluster labelled to the left of the heatmap.

**Figure 6:**
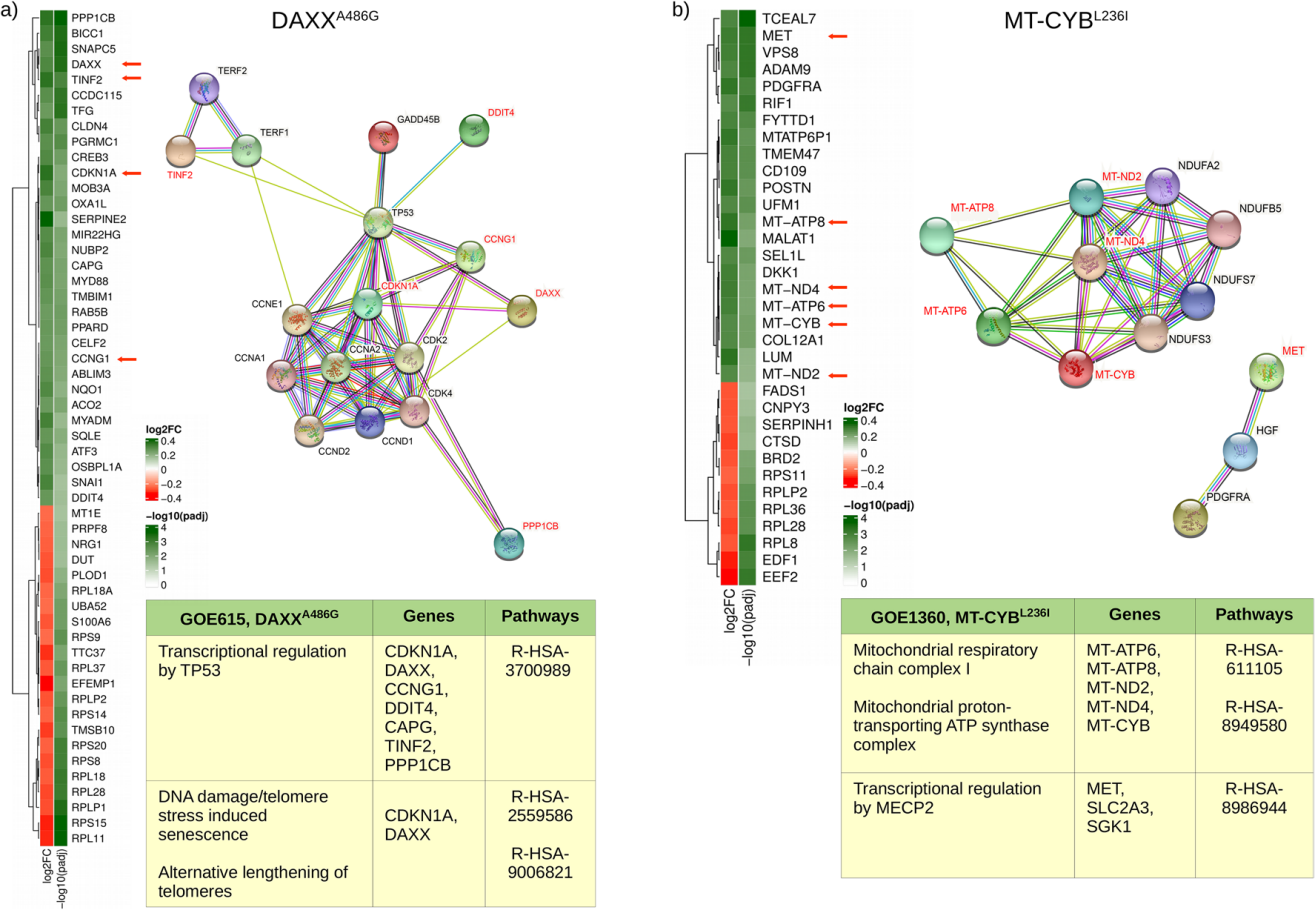
Variant-associated expression signatures in GOE615 and GOE1360. (**a**) Heatmaps showing log2FC and- log10 of adjusted *p* value for genes differentially expressed in mutant vs nonmutant cells for DAXX^A486G^ variant in sample GOE615 related to protein–protein interaction showing phenotype (StringDB) and pathways (Reactome) for this specific variant. (**b**) Heatmaps showing log2FC and- log10 of adjusted *p* value for genes differentially expressed in mutant vs non-mutant cells for MT-CYB^L236I^ variant in sample GOE1360 related to protein–protein interaction showing phenotype (StringDB) and pathways (Reactome) for this specific variant.

To this end, we highlighted mutant cells on the t-SNE projection of each of the six ageing-samples and evaluated markers related to hallmark mechanisms associated to each phenotype (Fig. 5a, b, c and Supplementary Fig. 5). Figure 6b displays variant DAXX^A486G^ (dbSNP rs146304558) which has been identified specifically in sample GOE615. The regulon shows the top 30 markers associated to this specific mutation, where three of them displayed significant regression to the variation with FDR < 0.05 (GADD45B, LOXL4 and ACKR3). Among GADD45B related pathways are p53 signaling^25,26^ and DNA damage ATM/ATR regulation of G1/S checkpoint^27^. This protein also associates with centromeres in G2 phase and in the cytoplasm the encoded protein may function to regulate apoptosis^28^.

Notably, four of the sample-specific variants found for GOE800, GOE1360, GOE486 and GOE1309 were located on mitochondrial genes exhibiting high-density mutation in each of the samples (Fig. 5c and Supplementary Fig. 5). For sample GOE800, variation MT-CO1^A122T^ (dbSNP rs202216551) showed high expression, with six markers showing significant regression to the variation (FDR < 0.05). Most of them are involved in hemostasis and formation of fibrin Clot (CD36, SERPINE2, F2R and F2RL2). Specifically, the overexpression of DAB1 was related to Spinocerebellar ataxia 37, epilepsy and involved in the lipoprotein metabolism^29^(Supplementary Fig. 5).

Variation MT-CYB^L236^^I^ (dbSNP rs193302994), found specifically in sample GOE1360, displayed 27 markers as significantly regulated (FDR < 0.05) in the heatmap of Fig. 5c. Many of those play a role in degradation of the extracellular matrix and collagen degradation (HTRA, LTBP2, COL11A1, ITGA1, MMP14, CTSD, COL11A1), transcriptional regulation by MECP2 (SLC2A3 and SGK1) and neutrophil degranulation (SLC2A3, LAMP1, DSP, GRN, CTSD). In particular, transcriptional regulation by MECP2 has been described in Rett syndrome as rare disease, but still one of the most abundant causes for intellectual disability in females^30^. The type and severity of symptoms are individually highly different. The most important action of MECP2 is regulating epigenetic imprinting and chromatin condensation^30,31^.

In order to understand how different biological pathways explain genetic regulation in association to phenotypes, we used the scRNA-seq data and performed differential expression analysis between cells exhibiting particular variations (mutant cells) and those who do not (non-mutant cells). We specifically performed this analysis on GOE1360 and GOE615 cells containing the MT-CYB^L236I^ and DAXX^A486G^, respectively (Fig. 6a, b). While in itself a useful method to inspect the effect of variations in cells on gene expressions, we expect its results to coincide with those of the regulon analysis as displayed in Figs. 5b and c.

For sample GOE615, the differential expression analysis performed between mutant and non-mutant cells for the variant DAXX^A486G^ displayed a significant overlap of genes involved in molecular pathways found to be associated to DAXX^A486G^ regulon-significant genes. The most prominent gene found to be over-expressed in mutant cells from sample GOE615, affecting the TP53 signalling at multiple levels, is the CDKN1A. Besides CDKN1A, the up-regulation of CCNG1, DDIT4, CAPG, TINF2, PPP1CB and DAXX result in enriched pathways for TP53 regulating transcription^32^. Moreover, the overexpression of CDKN1A is linked to TP53-dependent G1/S DNA damage checkpoint; TP53 regulating transcription of cell cycle genes; TP53-dependent G1 DNA damage response and TP53 regulating transcription of genes involved in G1 cell cycle arrest^33^. Notably, the DAXX gene itself was found to be over-expressed exclusively in sample GOE615, underlying the link from genetic variation to this specific progeria sample phenotype (Fig. 6a).

Mutations in the DAXX gene have been found frequently in both telomerase-positive and alternative lengthening of telomeres (ALT) cells. Endogenous DAXX can localize to Cajal bodies, associated with the telomerase inducing DNA damage/telomere and ALT^34,35^.

In addition, when comparing the 27 significant regulon markers for MT-CYB^L236I^ with the genes over-expressed in mutant cells for the same mutation, it was evident that most of those genes participate in the same pathways; suggesting concordance in both analyses. Most of the induced genes are related to degradation of the extracellular matrix and specifically, transcriptional regulation by MECP2 via MET affecting diseases of signal transduction and sex-specific expression in autism and Rett syndrome^36^. Interestingly, five mitochondrial genes were found regulated by this mutation (MT-ATP6, MT-ATP8, MT-ND2, MT-ND4 and MT-CYB), resulting in enriched pathways, “mitochondrial respiratory chain complex I”, “mitochondrial proton-transporting ATP synthase complex” and “mitochondrial respirasome” (Fig. 6b). As described above for DAXX gene, MT-CYB itself appears to be over-expressed only for sample GOE1360, providing a consistent evidence of linking variation heterogeneity to the phenotype for sample GOE1360. Mutations in MT-CYB have been reported to cause isolated complex III deficiency leading to a variety of symptoms including failure to thrive, exercise intolerance, cardiomyopathy and encephalomyopathy^37^. Some phenotypic variability in the gene has been reported in patients with additional complications of hearing loss, visual impairment, brain atrophy, cardiomyopathy and gastroparesis^38^.

It is worth noting that the deregulated genes between mutant and non-mutants cells for samples GOE615 and GOE1360 discussed in Fig. 6 connecting genetic regulation to phenotype within the same cell were also found as markers in the ICELL8 and CellenONE-ICELL8 data, demonstrating biological concordance with the most relevant genes described in this study (Supplementary Fig. 7).

## Discussion

In this study, we systematically compared the performance of two approaches for single-cell RNA-seq in primary dermal fibroblast cells by using a full-length chemistry, demonstrating its utility in multi-omics analysis such as the ability of a genotype to show phenotype diversity.

We demonstrated that the combination of the two different platforms, CellenONE for cell dispensing and cell documentation and ICELL8 for fully automated RT and library preparation, improves significantly the isolation and selection of single cells which is the most critical step for obtaining transcriptome and genome information from individual cells. Due to the exclusion of artifacts such as cell doublets and debris through image analysis prior to sequencing, computational filtering methods become unnecessary. One such computational routine is the doublet cell detection, which is executed based on a) excluding cell barcodes with unusually high numbers of detected transcripts, and b) reliance on manually curated data to exclude cell clusters that co-express marker genes in distinct cell types^39^. This method performs poorly as it assumes that cells contain similar amounts of RNA, when in reality samples with diverse cell types or cells in different cell cycle stages are expected to have a wide range in the number of transcripts per cell. Furthermore, it requires expert knowledge and careful annotation of the data to find co-expressed marker genes in different cell types^40^. For those reasons, it is more beneficial to assess the presence of such artifacts in single cell sequencing during the experimental step rather than by computational approaches.

The next improvement of the novel approach is the performance of full-length based chemistry on a high-throughput system. While previous techniques have been employing full-length sequencing for single-cells, but on a much smaller scale^9,13^, here we improved the capturing rate of single cells to allow the investigation of more than 3,300 cells per run after executing QC filtering on one ICELL8 5,184 nano-well chip.

The utilisation of full-length chemistry in the novel approach has yielded an increase in detection of intergenic and intronic sequences, as well as non-coding RNAs, especially long intergenic non-coding RNAs (lincRNAs). When comparing the novel approach to the ICELL8 platform, the former was found to have 10,725 expressed (more than 1 read) lincRNAs after QC filtering, while the latter had 3,336 expressed lincRNAs after QC filtering across all samples. The importance of this discovery is particularly for the evaluation of lincRNAs together with coding genes in disease, which belongs to the cutting-edge strategies for current state-of-the-art analysis in the transcriptomic context. Therefore, having a methodology, which allows us to determine the expression of lincRNAs in single cells, will thus be beneficial for understanding physiological and pathological processes.

Beyond the technical improvements, we have demonstrated the applicability of the novel approach and benefit of the full-length chemistry to clinical research, connecting genetic regulation (variant detection) to phenotype (gene expression) within the same cell. To achieve this, we have used the subsequent workflow: (1) studying transcriptional heterogeneity (Fig. 4), (2) finding sample-specific variants, (3) associating the top variants with transcriptional markers using regulon analysis (Fig. 5b and c), and (4) corroborating transcriptional heterogeneity by differential expression analysis of mutated vs. non-mutated cells based on the top variants (Fig. 6). Following the workflow yielded a partial description of the phenotype diversity.

It is important to note, that progeria is not fully described in the literature in terms of transcriptional markers. Nevertheless, we found high concordance in genes and pathways involved in biological processes and cellular hallmarks of ageing diseases when transcriptional and mutational heterogeneity were combined. For instance, sample GOE615 expressed mainly within clusters 4 and 3 in Figs. 4c and e shows transcriptional regulation of the TP53-related pathways (cell death signalling and cellular response to stress). These pathways were also found enriched for the GOE615 specific DAXX^A486G^ variant when differential expression analysis of mutated vs non-mutated cells was performed (Fig. 6a). Moreover, a comparable association is reported for sample GOE1360, where clusters 5 and 2 in Figs. 4c and e were affiliated to the MET activation pathway. The gene MET itself appears deregulated from the differential expression analysis for the variant MT-CYB^L236I^ (Fig. 6b).

In this study, we identified between 94 and 756 variants for each sample while reporting only the top ones, thus explaining a fraction of the phenotype diversity. In order to expand on it, it is necessary to evaluate many variants at once according to their significance to provide a better score on their relevant connection to transcriptional markers. Unfortunately, bioinformatic tools for that purpose are not available yet. Therefore, developing such tools will be essential for a comprehensive understanding of diseases in general when applying similar full-length scRNA-Seq methods in the future.

## Methods

### Study design

Eight primary dermal fibroblast cell cultures were used for the comparison of both cell-preparation approaches (ICELL8 library preparation and CellenONE cell extraction combined with ICELL8 library preparation).

### Cell culture of dermal Fibroblasts

Primary dermal fibroblast established from patient and respective controls were cultured in Dulbecco’s modified Eagle medium (DMEM, Gibco) supplemented with 10% fetal calf serum (FCS, Gibco), and antibiotics. Control samples GOE1303 and GOE1305 were purchased from Coriell Institute, New York, USA (GM02936C, GM00409D). Patient sample GOE1309 was purchased from Coriell Institute, New York, USA (GM02548D), while samples GOE1360, GOE800, GOE486, GOE247 and GOE615 were provided by our collaborators.

### Bulk RNA-sequencing

The start material (cell suspensions) used for single cell isolation were used for bulk sequencing (same source). Circa 100,000 cells were used for RNA extraction using the Trizol Reagent (Thermo Fisher) according to manufacturer’s recommendations. RNA-seq libraries were performed using 100 ng total RNA of a non-stranded RNA Seq (TruSeq RNA Library Preparation Cat. RS-122–2001). Libraries were sequenced on the Illumina HiSeq 4000 (SE; 1 × 50 bp; 30–35 Mio reads/sample).

### Full-length single-cell RNA-seq using the ICELL8 system

The Takara ICELL8 5,184 nano-well chip was used with the full-length SMART-Seq ICELL8 Reagent Kit. Cell suspensions were fluorescent-labelled with live/dead stain, Hoechst 33,342 and propidium iodide (NucBlue Cell Stain Reagent, Thermo Fisher Scientific) for 15 min prior to their dispensing into the Takara ICELL8 5,184 nano-well chip. CellSelect Software (Takara Bio) was used to visualize and select wells containing single and live cells. Next, cDNA was synthesized via oligo-dT priming in a one-step RT-PCR reaction. P5 indexing primers for subsequent library preparation were dispensed into all wells receiving a different index, in addition to Terra polymerase and reaction buffer. Transposase enzyme and reaction buffer (Tn5 mixture) were dispensed to selected wells. P7 indexing primers were dispensed to wells. Final Illumina libraries were amplified and pooled as they were extracted from the chip. Pooled libraries were purified and size selected using Agencourt AMPure XP magnetic beads (Beckman Coulter) to obtain an average library size of 500 bp. A typical yield for a library comprised of ~ 1,300 cells was ~ 15 nM. Libraries were sequenced on the HiSeq 4000 (Illumina) to obtain on average ~ 0.3 Mio reads per cell (SE; 50 bp).

### Full-length single-cell RNA-seq using the CellenONE-ICELL8 system

In the combined CellenONE-ICELL8 system, we used CellenONE to dispense single cells into the Takara ICELL8 5,184 nano-well chip. The cell dispensing was carried out on a temperature-controlled target holder in a humidity-controlled chamber. Placement and rotational errors were adjusted using the built-in “Find Target Reference Point (FTRP)” function. The cell suspension (300 cells/µl) was initially used to create a mapping that determine the cell size parameters based on cell types. For isolation of single human dermal fibroblasts, the following parameters were used: diameter 20–60 µm, elongation and circularity 1–1.5 µm excluding doublets and debris. After single cell dispensing, the Takara ICELL8 5,184 nano-well chip was sealed and centrifuged. Next, the chip was placed in the iCELL8 cx system and proceeded with the RT and library preparation as described in the section above.

### Pre-processing of data from CellenONE-ICELL8

Raw sequencing files (bcl) were converted into a single fastq file using Illumina bcl2fastq software (v2.20.0.422) for each method. Each fastq file was de-multiplexed and analyzed using Cogent NGS analysis pipeline (CogentAP) from Takara Bio (v1.0). In brief, “cogent demux” wrapper function was used to allocate the reads to the cells based on the cell barcodes provided in the well-list files. Subsequently, “cogent analyze” wrapper function performed a preliminary analysis, including: (1) read trimming with cutadapt^41^(version 3.2); (2) genome alignment to Homo sapiens genome GRCh38 using STAR^42^(version 2.7.2a); (3) read counting for exonic, genomic and mitochondrial regions in Homo sapiens genes from ENSEMBL gene annotation version 103 (https://www.ensembl.org/Homo_sapiens/Info/Index) using featureCounts^43^(version 2.0.1); and (4) summarising the gene counts into gene matrices with number of reads expressed for each cell in each gene. Raw gene matrices underwent quality-control (QC) filtering for cells and genes using the following parameters: (a) for cells, only those with at least 10,000 reads associated to at least 300 different genes, and (b) for genes, only those containing at least 100 reads mapped to them from at least 3 different cells.

### Gene marker detection

To determine gene markers expressed in specific samples, QC-filtered read count matrix was used as input to determine which genes were differentially expressed between cells from a particular sample and all other cells using Wilcoxon Rank Sum and Signed Rank Test, with* p* values adjusted using Benjamini–Hochberg method. For the individual differential expression results, a rank score was calculated for each gene using the formula $rank=-log10\left(pval\right)*log2FC$, where pval is the raw* p* value and log_2_FC is the log_2_ fold-change.

### Bulk RNA processing

Samples sequenced with bulk RNA-seq were analysed with the same tools as used for single-cell RNA-seq (de-multiplexing with bcl2fastq, read trimming with cutadapt^41^, genome alignment to Homo sapiens genome GRCh38 using STAR^42^ and read counting to exonic regions using featureCounts^43^ with gene annotation version 103 from ENSEMBL).

### Correlations of scRNA datasets and bulk RNA samples

Single-cell datasets were correlated for each sample individually for a select subset of genes. In brief, the markers in each sample from both scRNA-seq approaches were determined. The absolute rank scores from both methods were summed for each sample individually, and the top 100 markers with highest absolute rank scores were used to correlate the ICELL8 and CellenONE-ICELL8 datasets using the Pearson correlation. Moreover, single-cell dataset pseudo-counts were calculated as the sum counts for all genes in cells from each particular sample, and the resulting pseudo-counts were combined with the bulk RNA-seq samples to a single gene matrix, normalized using scale sizes calculated by DESeq2^44^(version 1.32.0) and correlated for the same markers.

### Unsupervised cell cluster

Using the CogentDS v1.0 R package, dimensionality reduction and visualization were performed with the t-SNE algorithm using the optimal principal components (PCs) chosen from elbow plots. In addition, unsupervised graph-based clustering was performed in order to group cells into particular clusters using CogentDS.

For each graph-based clustering, gene markers were detected, and the top 30 genes were plotted in a heatmap.

### Variant calling in scRNA and bulk data

For the scRNA-seq data, aligned reads were separated based on their sample affiliation into 8 different alignment files, and variants were identified in those alignment files, as well as alignment files for bulk RNA samples, using the Genome Analysis Toolkit^45^(GATK v4.1.927). Using in-house R scripts utilizing the R package VariantAnnotation^46^(v1.38), the VCF files were merged together, and for each patient sample (GOE1360, GOE800, GOE486, GOE247, GOE615 and GOE1309), sample-specific scRNA-seq variants corroborated by bulk data and with a high variant and total depths were retrieved.

### Expression signatures of target variants

For each sample, a single sample-specific variation was selected, such that it had a maximum variant depth, as well as being non-synonymous. For the target variations, the percentages of cells containing the mutation out of all cells in each sample were calculated, followed by a multiple regression analysis between average gene expressions per sample and the mutation percentages in each sample. The regression was calculated with the formula:

$${E}_{i}={x}_{i}+{y}_{i}m+{z}_{i}g$$

where ${E}_{i}$is the normalized average expression of each tested gene i, ${z}_{i}g$ are the normalized average expressions of the gene affiliated with the target mutation per sample, and ${y}_{i}m$ are the percentages of mutant cells, for which the* p* value was calculated. From the regression analysis,* p* values were calculated for all genes found to be markers in at least one sample, and corrected using the Benjamini-Hochberg. The normalized expressions of all QC-filtered cells were plotted in a heatmap for the top 30 genes with lowest adjusted* p* values.

### Deregulated genes in mutant cells

Using an in-house R script, the reads used to call each of the target variants were affiliated with their specific cell, such that cells with variation-containing reads were deemed “mutant cells” and others “non-mutant cells”. Similar to the “Gene marker detection” method mentioned above, Wilcoxon Rank Sum and Signed Rank Test was used to find deregulated genes between mutant and non-mutant cells. The resulting genes were filtered for the initial value of absolute log_2_ fold-change >  = 0.25 (discarding small differences in expressions between cell groups) to allow a more lenient adjustment of the* p* values using Benjamini–Hochberg method. Finally, the up- and downregulated genes were inserted into STRINGDB version 11.0b^47^ and REACTOME version 77^48^ to uncover how the deregulated genes are connected to each other and what ontologies and/or pathways they belong to.

## Supplementary Information

Supplementary Information 1.

Supplementary Information 2.

## Data Availability

All sequencing data has been deposited in the Gene Expression Omnibus (GEO) at GSE179211. Any other relevant data are available from the authors upon reasonable request. Scripts used to analyse the data and generate the figures and tables in this paper can be found on GitHub (https://github.com/UKHG-NIG/single-cell-cellenion-icell8).
