# Supplementary material for: A novel single-cell RNA-sequencing approach and its applicability connecting genotype to phenotype in ageing disease
Source: Sci Rep. 2022 Mar 8;12:4091. doi: 10.1038/s41598-022-07874-1 (PMC8904555; doi:10.1038/s41598-022-07874-1)
Supplement: Supplementary file 1 — Supplementary Information 1. [file 41598_2022_7874_MOESM1_ESM.pdf]

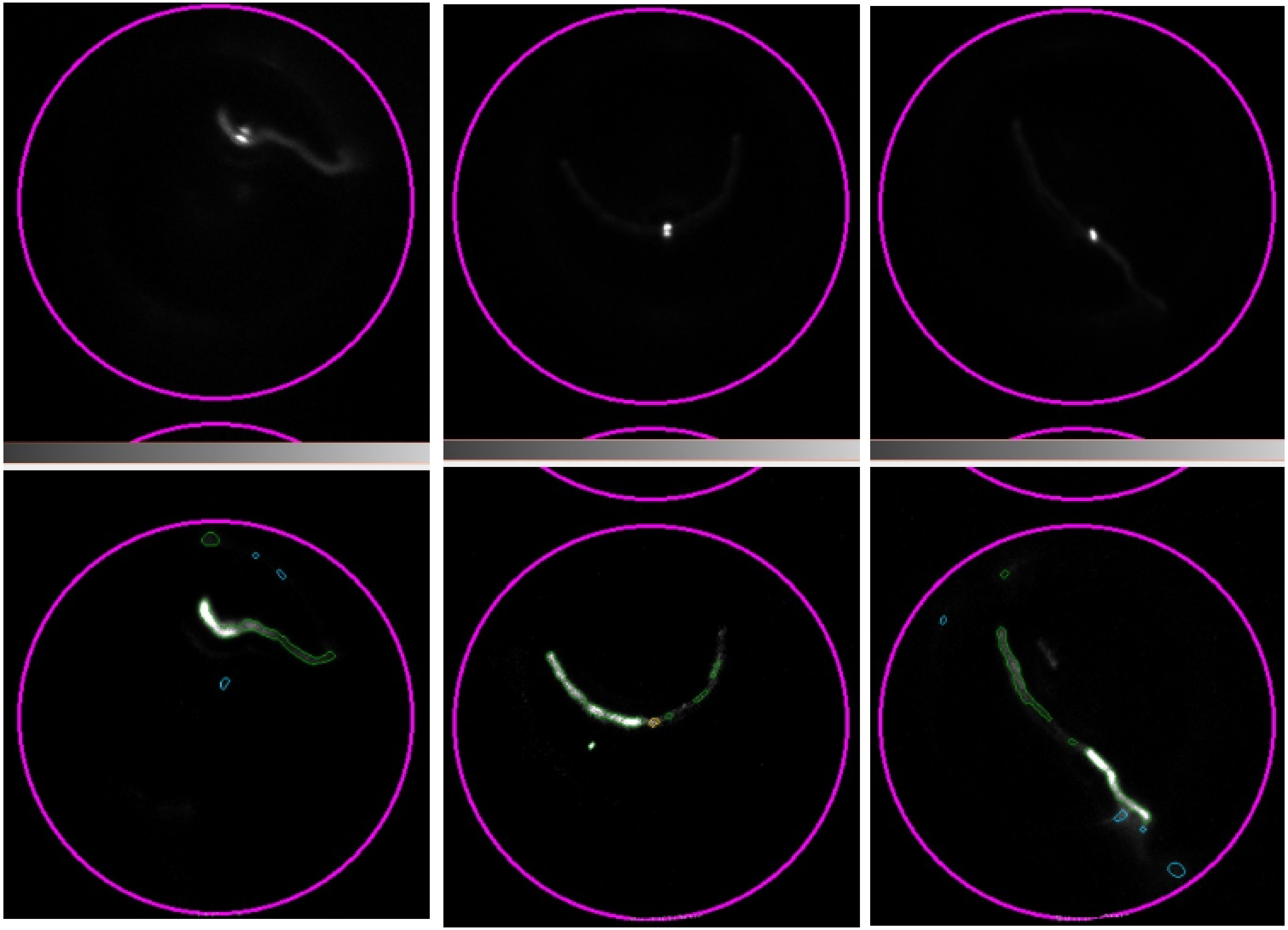

Supplementary Figure 1: dispensing of Schwann cells exhibiting axonic regrowing forms and sizing of 200-500 µm into a Takara ICELL8 5,184 nano-well chip. Cells were fluorescent-labelled with live/dead stain, Hoechst 33342 and propidium iodide (NucBlue Cell Stain Reagent, Thermo Fisher Scientific). Living and single cells were visualized and selected using the CellSelect Software (Takara Bio).

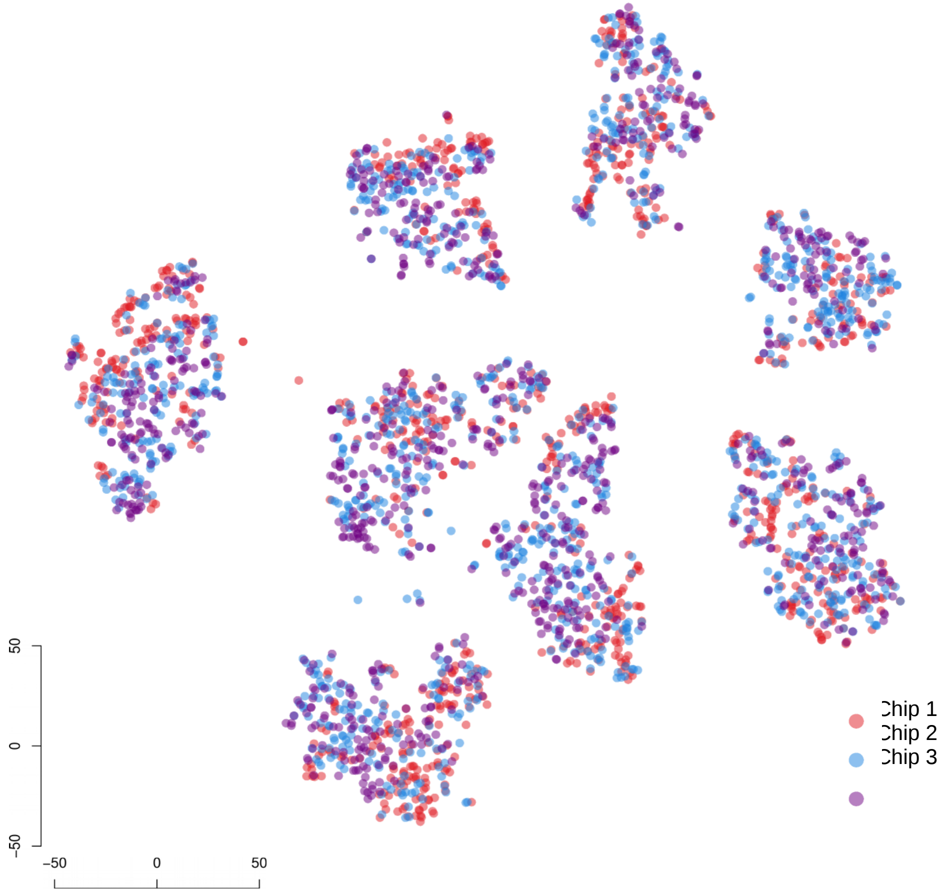

Supplementary Figure 2: t-SNE visualization of the three ICELL8 chips, showing how single cells from all 3 chips overlap with each other and showing no batch effect between them.

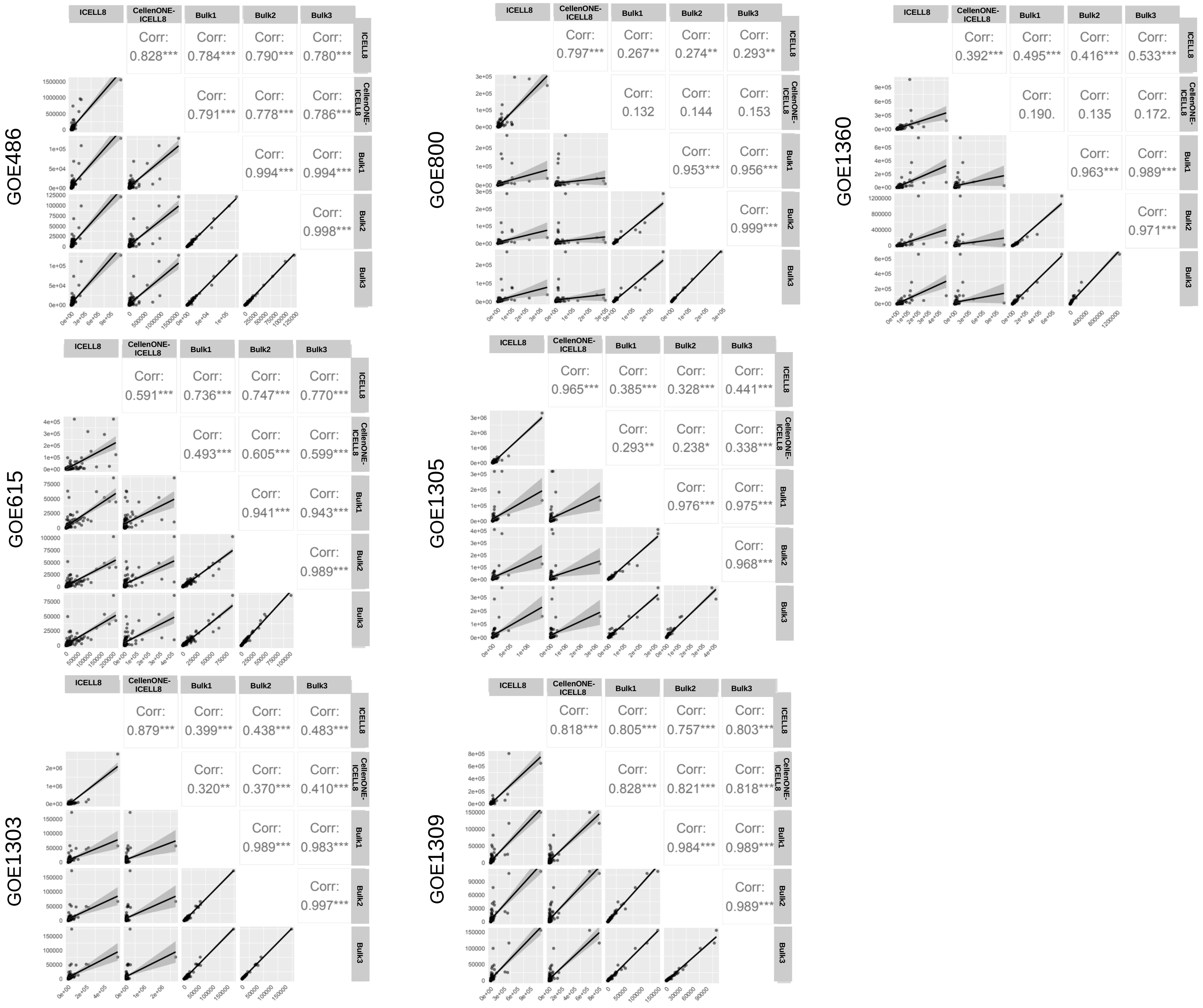

Supplementary Figure 3: Correlations of normalized expressions of the top 100 markers from samples in the scRNA-Seq platforms and bulk RNA-seq datasets, with the correlation coefficients indicated in the upper triangle.

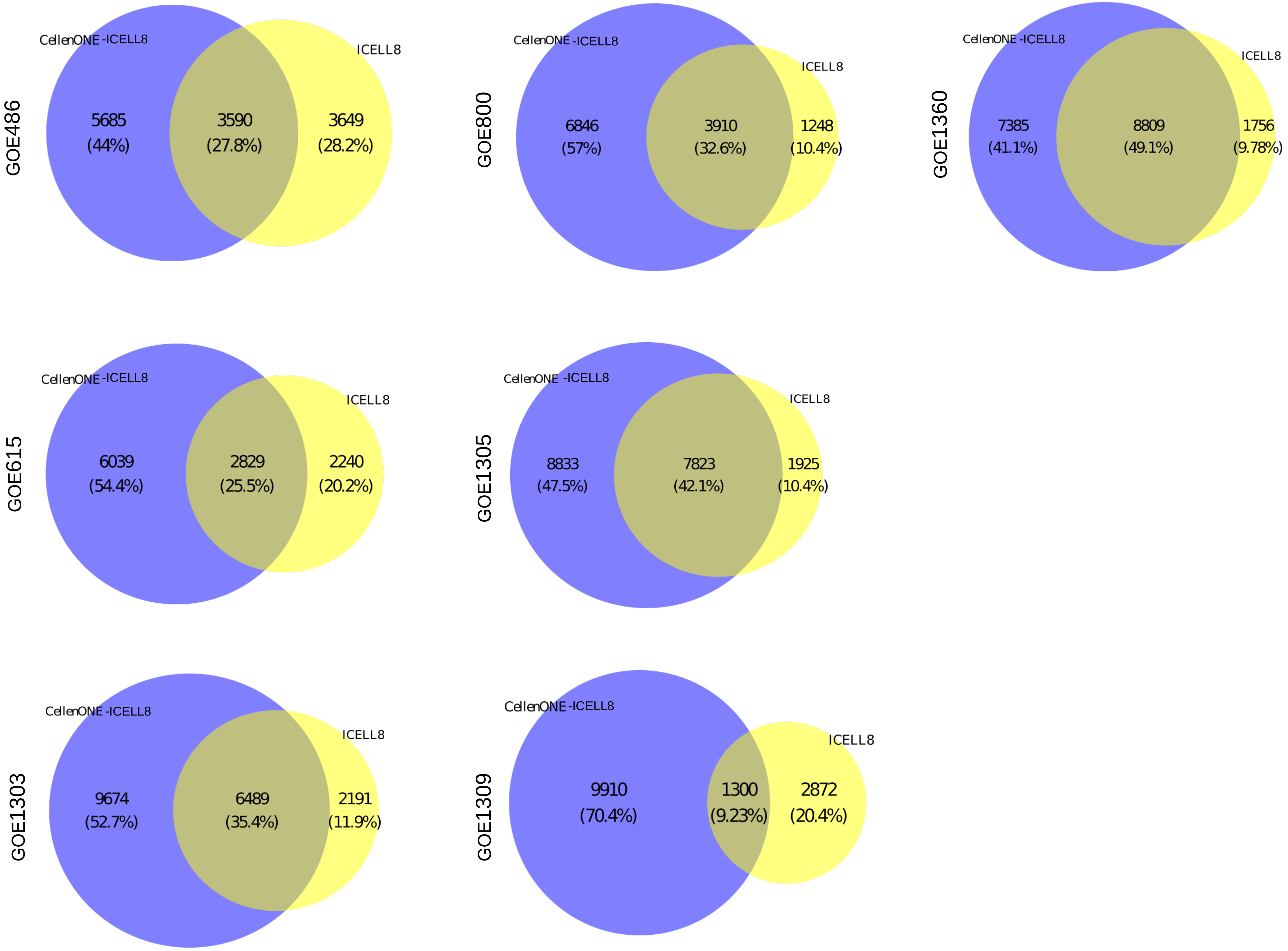

Supplementary Figure 4: Venn diagram of all significant markers detected for samples in both scRNA-Seq platforms.

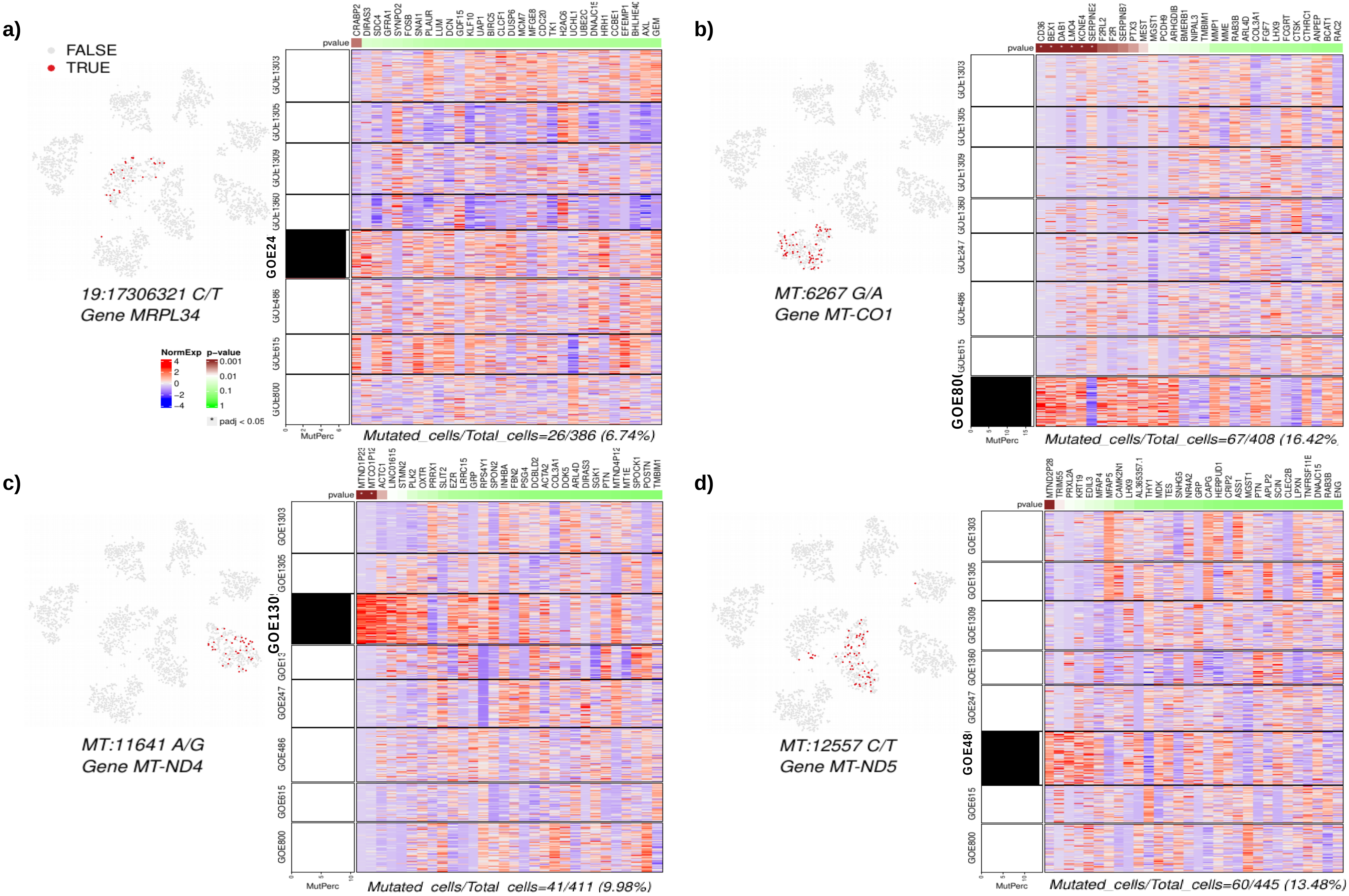

Supplementary Figure 5: Analysis of mutational heterogeneity for samples (a) GOE247, (b) GOE800, (c) GOE1309 and (d) GOE486. t-SNE plots show non-uniformly distributed variant-expressing cells in red. The heatmap displays the scaled, normalized gene expressions of the top 30 markers with highest adjusted p-values from the regression of the gene expressions to the mutation rate (p-values shown in top bar), and a bar graph on the letft shows mutant cell fraction in each cluster labelled to the left of the heatmap.

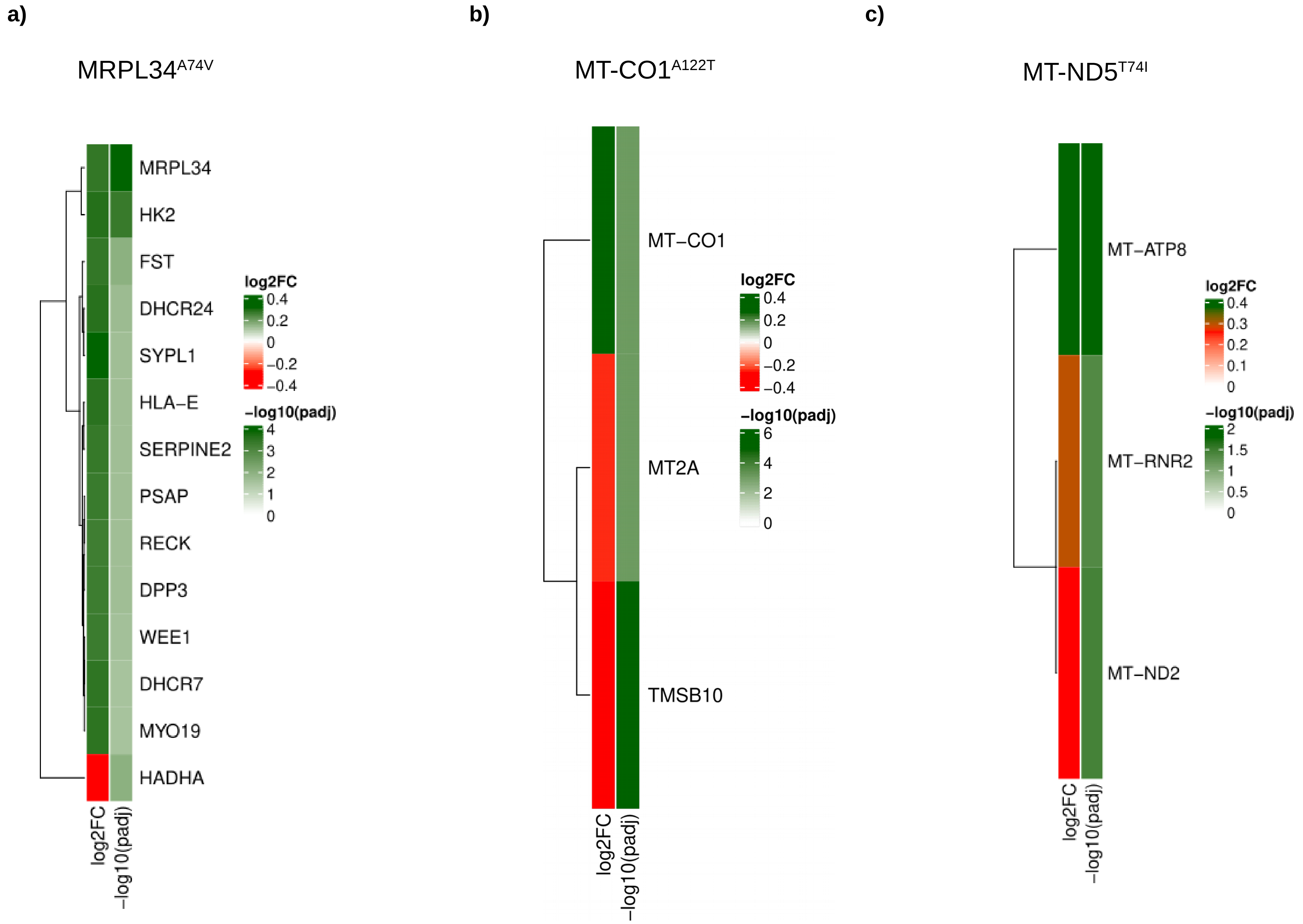

Supplementary Figure 6: Variant-associated expression signatures in samples (a) GOE247, (b) GOE800 and (c) GOE486. Heatmaps showing log_2FC and -log_{10} of adjusted p-value for differentially expressed genes between mutant and non-mutant cells for (a) MRPL34^{A74V} variant in sample GOE247 (b) MT-CO1^{A122T} variant in sample GOE800 and (c) MT-ND5^{T74I} variant in sample GOE486.

a)

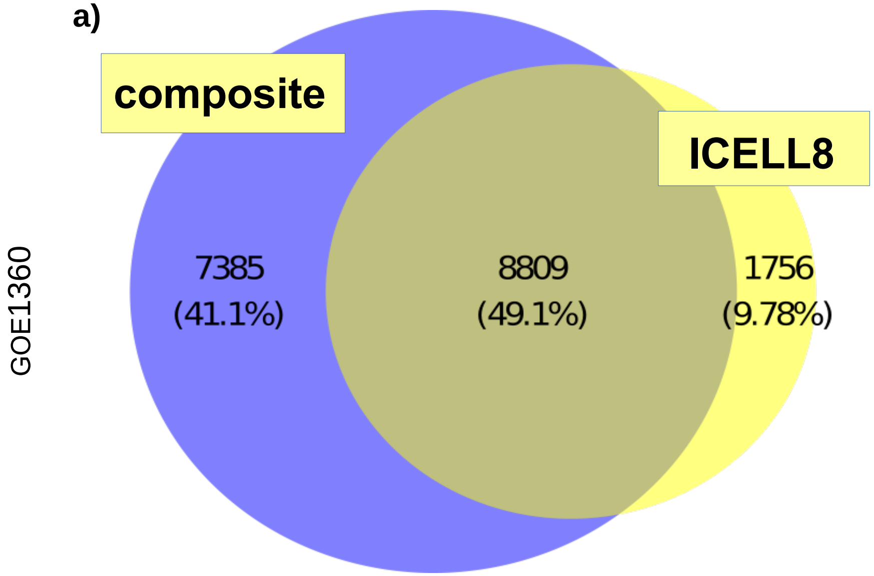

b)

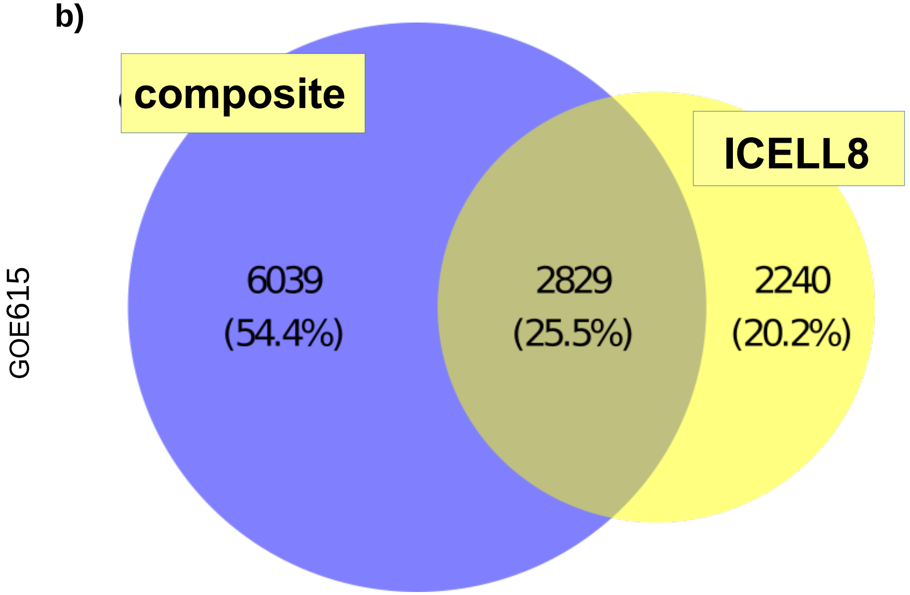

GOE1360, MT-CYB^{L236I}	Composite	ICELL8
Mitochondrial respiratory chain complex I<br><br>Mitochondrial proton-transporting ATP synthase complex	MT-ATP6,<br>MT-ATP8,<br>MT-ND2,<br>MT-ND4,<br>MT-CYB	MT-ATP6,<br>MT-ATP8,<br>MT-ND2,<br>MT-ND4,<br>MT-CYB
Transcriptional regulation by MECP2	MET,<br>SLC2A3,<br>SGK1	MET,<br>SLC2A3,<br>SGK1

GOE615, DAXX^{A486G}	Composite	ICELL8
Transcriptional regulation by TP53	CDKN1A,<br>DAXX,<br>CCNG1,<br>DDIT4,<br>CAPG,<br>TINF2,<br>PPP1CB	CDKN1A,<br>DAXX,<br>CCNG1,<br>DDIT4,<br>CAPG,<br>TINF2,<br>PPP1CB
DNA damage/telomere stress induced senescence<br><br>Alternative lengthening of telomeres	CDKN1A,<br>DAXX	CDKN1A,<br>DAXX

Supplementary Figure 7: Venn diagram of all significant markers detected and regulated in both scRNA-Seq approaches indicating the most relevant genes connecting genetic regulation to phenotype found in both scRNA-Seq approaches (a) sample GOE1360, (b) sample GOE615
